# The 10 sea urchin receptor for egg jelly proteins (SpREJ) are members of the polycystic kidney disease-1 (PKD1) family

**DOI:** 10.1186/1471-2164-8-235

**Published:** 2007-07-13

**Authors:** H Jayantha Gunaratne, Gary W Moy, Masashi Kinukawa, Shinji Miyata, Silvia A Mah, Victor D Vacquier

**Affiliations:** 1 Center for Marine Biotechnology and Biomedicine, Scripps Institution of Oceanography, University of California San Diego, La Jolla, CA 92093-0202, USA; 2 Jacobs School of Engineering, University of California San Diego, La Jolla, CA 92093-0403, USA; 3 Experimental Therapeutic Center, Institute of Molecular and Cell Biology, 61 Biopolis Drive, Proteos, 138673, Singapore

## Abstract

**Background:**

Mutations in the human polycystic kidney disease-1 (*hPKD1*) gene result in ~85% of cases of autosomal dominant polycystic kidney disease, the most frequent human monogenic disease. PKD1 proteins are large multidomain proteins involved in a variety of signal transduction mechanisms. Obtaining more information about members of the PKD1 family will help to clarify their functions. Humans have five hPKD1 proteins, whereas sea urchins have 10. The PKD1 proteins of the sea urchin, *Strongylocentrotus purpuratus*, are referred to as the Receptor for Egg Jelly, or SpREJ proteins. The SpREJ proteins form a subfamily within the PKD1 family. They frequently contain C-type lectin domains, PKD repeats, a REJ domain, a GPS domain, a PLAT/LH2 domain, 1–11 transmembrane segments and a C-terminal coiled-coil domain.

**Results:**

The 10 full-length SpREJ cDNA sequences were determined. The secondary structures of their deduced proteins were predicted and compared to the five human hPKD1 proteins. The genomic structures of the 10 SpREJs show low similarity to each other. All 10 SpREJs are transcribed in either embryos or adult tissues. SpREJs show distinct patterns of expression during embryogenesis. Adult tissues show tissue-specific patterns of SpREJ expression.

**Conclusion:**

Possession of a REJ domain of about 600 residues defines this family. Except for SpREJ1 and 3, that are thought to be associated with the sperm acrosome reaction, the functions of the other SpREJ proteins remain unknown. The sea urchin genome is one-fourth the size of the human genome, but sea urchins have 10 SpREJ proteins, whereas humans have five. Determination of the tissue specific function of each of these proteins will be of interest to those studying echinoderm development. Sea urchins are basal deuterostomes, the line of evolution leading to the vertebrates. The study of individual PKD1 proteins will increase our knowledge of the importance of this gene family.

## Background

The sea urchin is a model animal for cell and developmental biology and genomics. As a basal deuterostome, it provides an out-group for the chordates, and thus insights into vertebrate genome evolution. The genome of the purple sea urchin, *Strongylocentrotus purpuratus *(Sp), is one-quarter the size of the human genome and encodes ~23,300 genes [1], the majority having vertebrate orthologs. Sea urchin genes are closer to human and mouse than to other invertebrate models such as *Drosophila *and *C. elegans*. For example, the number of reciprocal pairs of genes between sea urchin and mouse is about 50% greater than between sea urchin and *Drosophila *[1]. The sea urchin lacks four of the human kinase subfamilies, while *Drosophila *lacks 20 and nematodes 30 [1,2]. Regarding gene conservation, 11 of the 13 known animal *Wnt *genes are present in the sea urchin [3]. The sea urchin has ~100 demonstrated human disease gene orthologs (Table S9, on line supplement to ref.1). The functions of sea urchin disease gene orthologs are thus important to human medicine.

In our study of sea urchin sperm receptor proteins that bind egg jelly to trigger the exocytotic acrosome reaction [4], we have described four sperm plasma membrane proteins, named Sp Receptor for Egg Jelly 1–4 (SpREJ1-4) [5-8]. The first of these proteins, SpREJ1, is a heavily glycosylated, 210 kDa receptor that binds the fucose sulfate polymer of egg jelly to trigger the sperm acrosome reaction [5,9]. Monoclonal antibodies to SpREJ1 induce the acrosome reaction and compete with the fucose sulfate polymer [5]. SpREJ1 localizes to the plasma membrane covering the acrosomal vesicle and to the flagellar membrane [10]. The paralog, SpREJ2, localizes to the entire sperm plasma membrane, but is concentrated over the mitochondrion. It is thought to be intracellular because it does not label when intact cells are radioiodinated and it is not glycosylated [7]. In sperm, SpREJ3 (suREJ3) is only found in the plasma membrane over the sperm acrosomal vesicle [6]. SpREJ4 is structurally similar to SpREJ3, but is only found in the sperm flagellar membrane [8]. Homology searches with the four SpREJ proteins showed that they all contain a large domain, named the "Receptor for Egg Jelly" domain (REJ domain) that occurs in only one gene family, the polycystic kidney disease-1 (*PKD1*) family, found in both protostomes [11] and vertebrates [12,13]. The Pfam database (PF02010) defines the REJ domain as ~600 amino acids having six conserved Cys residues. Although SpREJ proteins are PKD1 family members, we prefer to use the SpREJ designation to be consistent with past publications [5-9].

In humans, the *hPKD1 *gene is expressed in many tissues in addition to kidneys and encodes a glycoprotein of 4,303 amino acids, also called hPKD1, or polycystin-1 (hPC1), much of which is extracellular and has the characteristics of a lectin-like, signal transduction molecule [12,13]. Mutations in hPKD1 cause ~85% of the cases of autosomal dominant polycystic kidney disease (ADPKD), probably the most common human monogenic disease with an incidence of > 1:1000, which is characterized by progressive development and enlargement of cysts, resulting in end-stage renal failure [12,13]. The other ~15% of ADPKD cases arise from mutations in hPKD2, or polycystin-2 (hPC2), a member of the TRP family that forms a cation channel when exogenously expressed [14,15]. hPKD1 and hPKD2 proteins are associated through coiled-coil domains in their carboxyl termini, and it is thought that hPKD1 regulates the activity of the hPKD2 cation channel [12,13] and also chloride transporters [16]. Recent evidence indicates that mammalian PKD1 is a mechanoreceptor, associated with the primary cilium of kidney tubule epithelial cells. The primary cilium senses fluid flow in the tubule and that in turn is thought to regulate the activity of the hPKD2 channel [11,12,17,18].

In addition to hPKD1, there are four other human orthologs: hPKDREJ, hPKD1L1, hPKD1L2 and hPKD1L3. hPKD1 protein is found in most human tissues. The intronless *hPKDREJ *is only expressed in mammalian testis [19] and associates with several TRP channel proteins and modulates G-protein signaling [20]. Mammalian PKDREJ, like sea urchin SpREJ3, is found only in the plasma membrane covering the sperm acrosomal vesicle [21]. Like hPKDREJ, SpREJ3 also associates with a sea urchin sperm TRP channel, SpPKD2 [22]. hPKD1-like-1 (hPKD1L1) has high expression levels in testis Leydig cells and heart [23]. hPKD1L2 is found in a long and short form, the short form beginning in exon 12 of the long form. The short form is expressed in heart and kidney and the long form in brain and testis [24]. hPKD1L3 associates with the TRP protein hPKD2L1, to form a cation channel in taste buds for the sensation of sour [25]. hPKD1L3 does not contain a REJ domain, but it is included in the PKD1 family because of other PKD1 homologous domains. hPKDREJ, and the three hPKD1L proteins all contain a cation channel domain made up by TM segments 6–11.

Searching the sea urchin genome [1] for homologs containing a REJ domain, yielded six novel *SpREJ *genes in addition to the four already known SpREJ cDNA sequences. The sequences of the six novel SpREJ cDNAs were determined and their secondary structures predicted. Here we compare all 10 SpREJ predicted proteins to the five human hPKD1 family proteins. Data are also presented on the levels of expression of the 10 *SpREJ *genes during embryogenesis and also in six adult sea urchin tissues.

## Results

### Cloning SpREJ cDNAs

Searching the sea urchin genome database with the four known SpREJ sequences identified the six unknown orthologs with REJ domains. All start and stop codons and all full-length cDNA sequences were obtained experimentally. All 10 genes were completely annotated and their major features determined (Table 1). Genome analysis shows that all 10 SpREJ genes are single copy.

**Table 1:** Features of the SpREJ genes and proteins

	Protein		Gene		
	
	Amino Acids	REJ^a^	kbp	exons	Scaffold_v2 number/s^b^
SpREJ1	1450	545	~25	20	54390, 189999
SpREJ2	1472	543	> 24	20	91722, 73980, 72263, 4298, 31499
SpREJ3	2681	549	~35	27	47463
SpREJ4	2829	571	> 50	24	54299, 53368
SpREJ5	3716	497	~46	41	40445
SpREJ6	3553	466	~48	31	53367
SpREJ7	3580	531	~89	52	87087
SpREJ8	3274	347	~28	30	713116
SpREJ9	2965	319	~31	29	28079
SpREJ10	2122	517	~20	20	87124

^a ^Number of amino acids of REJ domain

^b ^These numbers are from WGS assembly version 2.0, of June 15, 2006 [36].

### SpREJ protein features compared to human PKD1 orthologs

**Figure 1:**
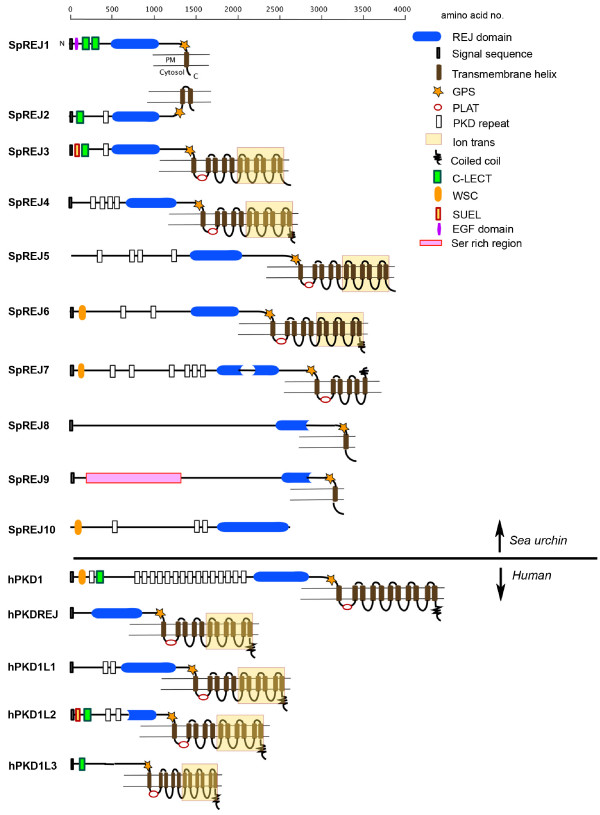
The PKD1 family of proteins as exemplified by the 10 SpREJs and five hPKD1 protein architectures. The predicted secondary structures are shown. Domain boundaries were taken from the Pfam database. The REJ domain is split into two sections in SpREJ7 and partial REJ domains occur in SpREJ8 and 9, and hPKD1L2. hPKD1L3 does not contain a REJ domain. Most of these predicted structures show a GPS domain upstream of transmembrane helix 1 (TM1) and a PLAT/LH2 domain immediately after the first TM.

Protein architectures based on secondary structure predictions of the 10 SpREJ proteins and the five human PKD1 proteins are shown in Figure 1. SpREJ10 is the only one in which a transmembrane segment (TM) is not predicted. SpREJs 1, 8 and 9 are predicted to have one TM, SpREJ2 two TM, SpREJ7 six TM, and SpREJ3-6, and all five human PKD1 proteins, 11 TM segments. The last six TM of SpREJ3-6, like the hPKD1L proteins, share homology with cation channels [12,13].

The REJ domain is found in four of the human PKD1 proteins. The fifth human PKD1 ortholog (PKD1L3) does not possess a REJ domain, however it is classified as a PKD1 protein because of having 11 putative TM, a GPS domain and a PLAT domain [26,27]. SpREJ8 and 9, and human hPKD1L2, all have partial REJ domains. SpREJ7 has a split REJ domain of 247 and 284 residues, with 428 non-REJ residues between the two REJ sections. All the other SpREJ proteins and hPKD1, hPKDREJ, hPKD1L1 have complete REJ domains with all six conserved Cys residues.

With the exception of SpREJ10, all these predicted sea urchin and human PKD1 family proteins contain a G-protein-coupled receptor cleavage site (GPS) upstream of the first TM. The GPS is where the protein chain is known to be cleaved in SpREJ3 [6] and human hPKD1 [28], yet both halves of the protein remain associated on the cell surface. It is assumed that all PKD1 family members are most likely cleaved at the GPS domain. The five human proteins and five of the predicted sea urchin proteins have a PLAT/LH2 domain (polycystin-1, lipoxygenase, alpha-toxin domain, also called a lipoxygenase homology 2 domain) immediately following the first TM [26,27]. The PLAT domain is always found intracellularly and supports the topology shown in Figure 1.

SpREJ2 is the only sea urchin protein that experimental data suggests is mainly intracellular because it is not labeled by vectoral radioiodination of the sperm surface and it does not appear to be glycosylated [7]. SpREJ1 becomes heavily labeled by vectoral iodination and it is 50% carbohydrate [5,10,29]. SpREJ2 and 7 are the only SpREJ proteins predicted to have both N- and C-termini on the same side of a membrane. Although only SpREJ4, 6 and 7 are predicted to have C-terminal coiled coils, all five human proteins show this motif that is involved in protein-protein interaction.

Carbohydrate recognition domains of ~120 amino acids (C-type lectin domains) are found in SpREJ1, 2, 3 and hPKD1 and hPKD1L2. There is one EGF domain in SpREJ1 [5], and one sea urchin egg lectin domain (SUEL) in SpREJ3 [6] and hPKD1L2. "PKD" repeats (50–70 residues) are found in SpREJ2-7 and 10 and hPKD1, hPKD1L1 and hPKD1L2. SpREJ6, 7 and 10 and human hPKD1 contain a WSC domain, which was originally identified in yeast cell wall integrity and stress response proteins [30]. In SpREJ9, there is a serine-rich region close to the N-terminus from Ser^173 ^to Ser^284 ^and Ser^528 ^to Ser^694 ^composed of 76% Ser residues. Hydropathy analysis shows that a signal sequence is present in all five human proteins and all SpREJs except SpREJ5. Multiple attempts to experimentally obtain a signal sequence for SpREJ5 failed. Furthermore, one could not be found in the predicted gene model.

Construction of phylogenetic trees using full-length sequences of the 10 sea urchin and five human proteins yielded branch nodes with low statistical support. The same negative results were obtained when only the REJ domains were used to construct trees. The only consistent trend was that SpREJ 3 and 4 branched close to hPKDREJ.

### Gene structure

The intron and exon numbers and scaffold positions of the 10 SpREJ genes were determined (Table 1). SpREJ1 is in three segments in two scaffolds, while SpREJ2 is in five scaffolds, thus with the current assembly it is difficult to compare these two genes. The REJ domains of these two proteins are 82% identical. Similarity in the downstream portions of SpREJ3 and 4 is clearly evident, as it is in the upstream portions of SpREJ5 compared to 6, and SpREJ8 compared to 9. Table 1 gives the number of exons per gene, total residues and predicted molecular masses of these proteins. The genomic sequence of SpREJ7 has 52 exons, which makes it the largest of the SpREJs. The low similarity of intron/exon numbers and lengths suggests that the duplication and subsequence differentiation of these genes is ancient.

### Expression of *SpREJ *genes in developing embryos

**Figure 2:**
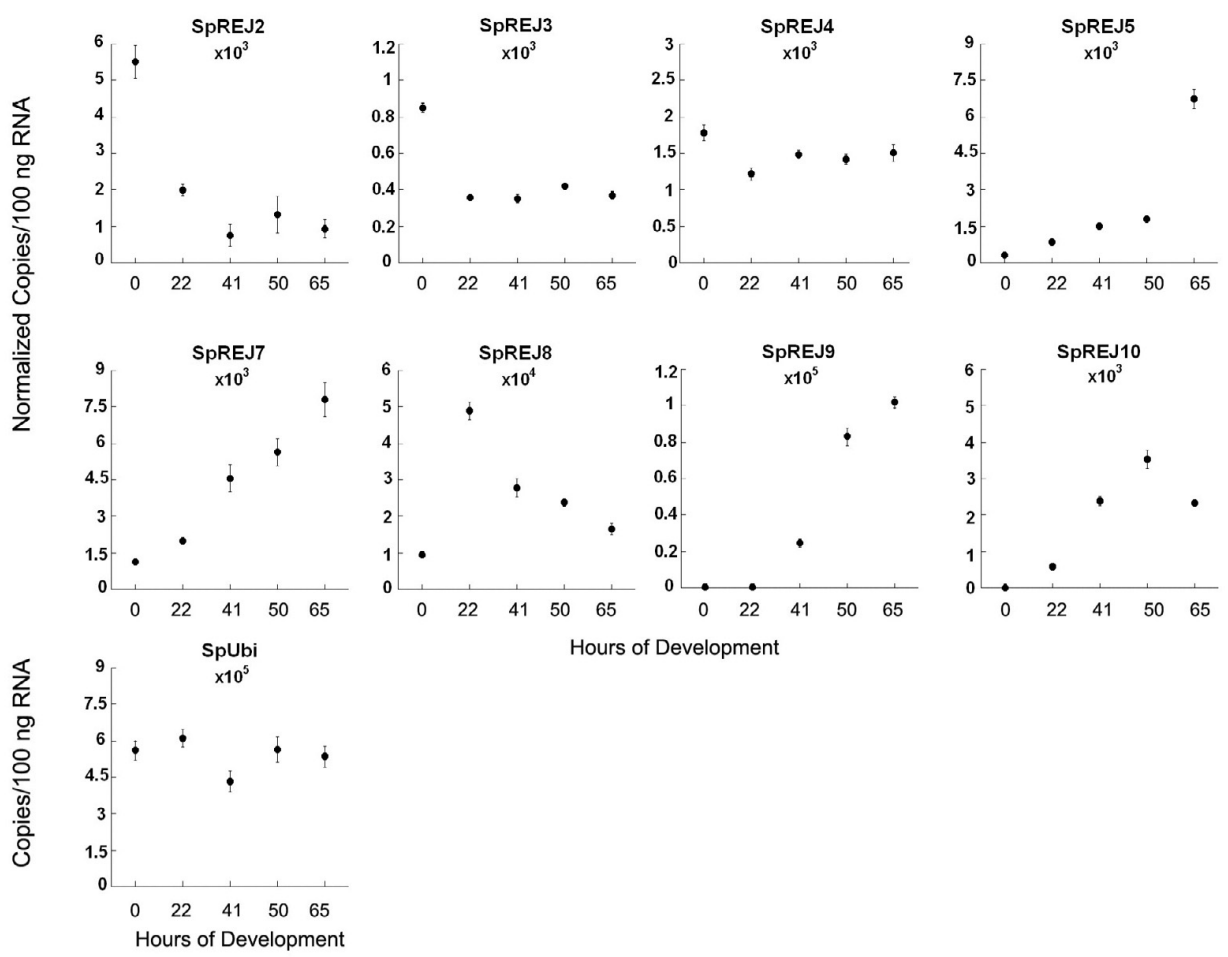
Expression of SpREJ transcripts during embryogenesis. Transcripts of SpREJ1 and 6 could not be detected in embryos. Transcript copy numbers per 100 ng of starting total RNA at each developmental stage for 8 SpREJs were first calculated. Transcript copy numbers were calculated from a standard curve generated in the same run and were normalized to the level of ubiquitin (SpUbi) transcripts at each developmental time. Final numbers are the averages of three replicas. There are about 50,000 transcripts per 100 ng total RNA of SpREJ8 at 22 hours of development. The standard error of each data point is shown (n = 3). Horizontal axes are hours of development at 16°C.

The expression of the 10 SpREJs at different embryonic stages was investigated using real-time quantitative PCR (Figure 2). Previous work on *S. purpuratus *showed that the ubiquitin transcript (SpUbi) levels are constant during early development, so that other transcripts can be normalized to ubiquitin (31). Under our experimental conditions, the expression of SpREJ1 and 6 were not detectable in early embryos. SpREJ2 is most abundant in eggs and decreases ~6-fold by 41 hours and then remains constant to 65 hours. SpREJ3 is also highest in eggs, but decreases about 60% to constant levels by 22 hours. SpREJ4 expression remains fairly constant from 0 to 65 hours. SpREJ5, 7, 9 and 10 show substantial increases in transcript number during development. SpREJ8 is the only one that increases sharply from 0 to 22 hours and then decreases sharply from 22 to 65 hours. Of all ten SpREJ transcripts, the greatest abundances are seen for SpREJ8 at 22 hours and SpREJ9 at 65 hours.

### Expression of *SpREJ *genes in adult sea urchin tissues

**Figure 3:**
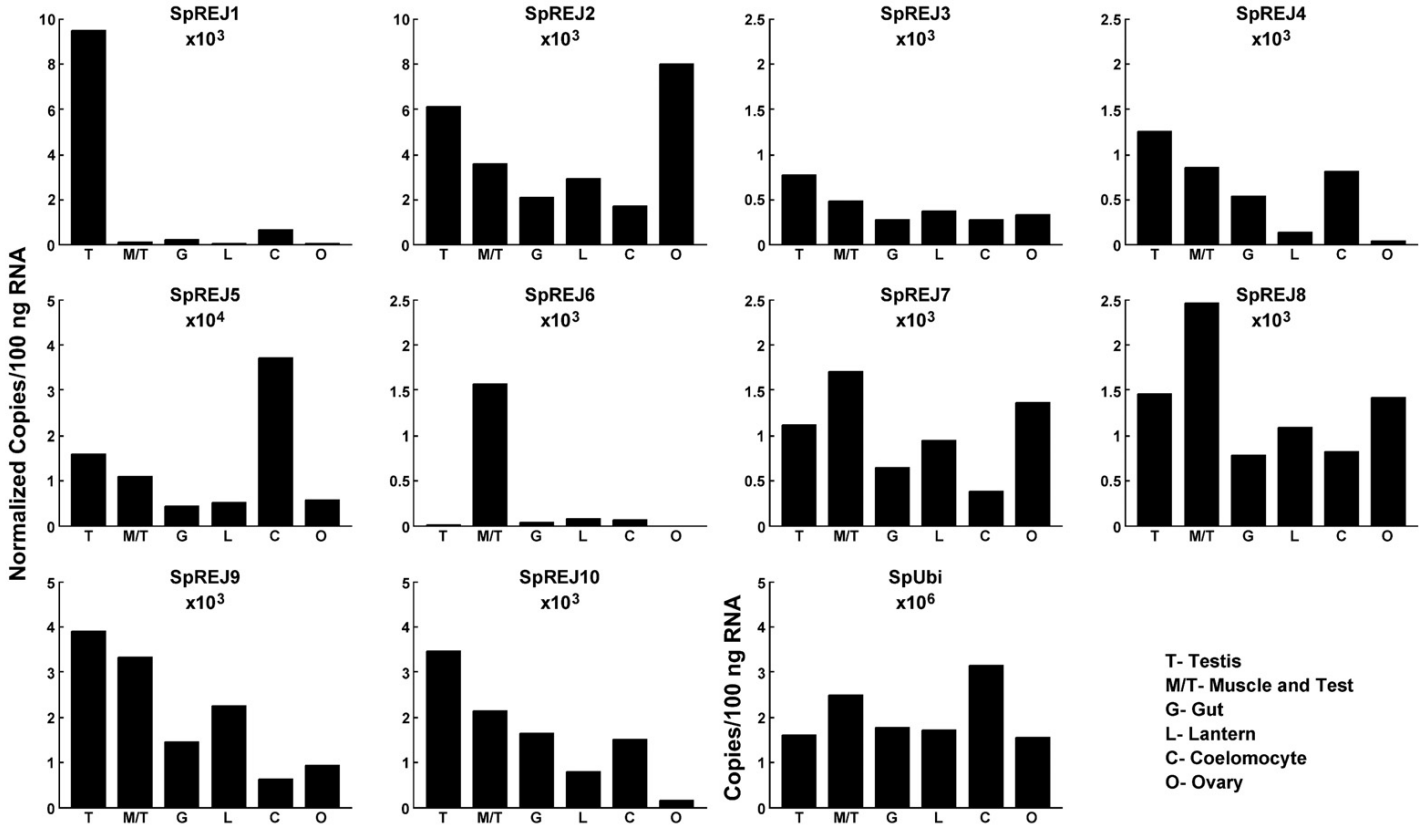
SpREJ transcript levels in six adult tissues. Transcript copy numbers per 100 ng of total RNA from each tissue was first calculated for all SpREJs. Transcript copy numbers were calculated from a standard curve generated in the same run and were normalized to the level of ubiquitin transcripts. It should be noted that the constancy of ubiquitin transcripts in adult tissues has not been determined. Final copy numbers were taken as the averages of duplicates. T, testis; O, ovary; M/T, muscle/test of body wall; G, gut; L, lantern (jaws, ligaments and muscles); C, coelomocytes (immunocytes).

RT-Q-PCR was used to determine the expression of *SpREJ *genes in six adult tissues (Figure 3). Testis, ovary, muscle/test (calcareous skeleton), gut, lantern (jaw muscles and ligaments) and coelomocytes (immunocytes in the body cavity fluid) were the six tissues that can be visibly separated from each other for RNA extraction. The range of ubiquitin transcript number is not known for adult tissues. Our data show that ubiquitin transcripts range from approximately 1.7 million per 100 ng total RNA in ovary, to 3.2 million per 100 ng RNA in coelomocytes. This might be expected because coelomocytes are highly phagocytic cells. Ubiquitin transcript levels are fairly similar in testis, gut, lantern and ovary. With the caveat regarding ubiquitin transcript levels stated, we did normalize the SpREJ transcript levels to ubiquitin. Relative to the other SpREJ transcripts, SpREJ1 is highly expressed only in testis [5] and the same is true for SpREJ6 expression in muscle/test. SpREJ1, 4, 6 and 10 have relatively low expression in adult ovary. SpREJ2, 3,6, 7 and 9 show low expression in coelomocytes. SpREJ6 is the only SpREJ that is weakly expressed in testis. To generalize, with the exception of SpREJ1 in testis and SpREJ6 in muscle/test, most *SpREJ *genes are expressed in easily measurable levels in these adult tissues. Each adult tissue shows a tissue-specific pattern of expression of the 10 SpREJ transcripts.

## Discussion

Whole genome sequences of model organisms permit the discovery of gene families involved in human disease. The sea urchin genome contains ~100 human disease gene orthologs (1). The functions of many of these orthologs remain unknown. Genome analysis of lower deuterostomes identifies disease gene orthologs for future functional studies. In this report we describe the sea urchin *SpREJ *gene family that are members of the larger *PKD1 *gene family, whose mutations in humans are associated with autosomal dominant polycystic kidney disease. SpREJ proteins are a subset of the PKD1 family because of possession of REJ, GPS and PLAT/LH2 domains. Our interest in the SpREJ proteins came from the observation that monoclonal antibodies to SpREJ1 increased intracellular calcium and induced the sea urchin sperm acrosome reaction [5]. When the protein sequence of SpREJ1 was deduced, the REJ domain appeared to match only one other protein, which was hPKD1. SpREJ2-4 were discovered next, 3 and 4 having 11TM and being similar to hPKD1 and hPKDREJ, whereas, SpREJ1 and 2 lacked this large TM domain and had either one or two TM. SpREJ1-3 are the only SpREJ proteins with CRDs. Sequencing the CRDs from six species shows that these lectin domains are subjected to positive Darwinian selection [32], indicating that they are probably important in gamete recognition [33]. hPKD1L2 and 1L3 each contain one CRD, but carbohydrate ligands binding these proteins remain unknown. The other sugar binding domain found in these proteins is the single sea urchin egg lectin domain (SUEL) in SpREJ3 and hPKD1L2. The SUEL lectin in the sea urchin egg has specificity for galactose [34].

hPKD1 is involved in mechanotransduction, regulation of cell growth, cell spreading and differentiation [12,13]. Four hPKD1 proteins and four SpREJ proteins contain cation channel domains. There are no experimental data for possible functions of the SpREJ5-10 proteins. To generalize, PKD1 family proteins are large transmembrane glycoproteins, which bind to smaller proteins to regulate signal transduction pathways and ion channel activities. SpREJ1-4 are definitely sperm plasma membrane proteins that are concentrated in different parts of the sea urchin sperm [5-8,10]. However, the membrane compartments of SpREJ5-10 in gametes, embryos and adult tissues remain unknown. With the exception of SpREJ10, all SpREJ and hPKD1 proteins have putative TM helices.

"PKD repeats", which are 50–70 residues and have similarity to the immunoglobulin fold, are present in some of the sea urchin and human proteins. hPKD1 has 16 PKD repeats, 15 of them in tandem. With the exception of hPKD1L3, all human and sea urchin proteins have either complete or partial REJ domains. The SpREJ and PKD1 family proteins are the only proteins known to contain both PKD repeats and REJ domains. Experimental work suggests that PKD repeats and REJ domains might have the same function in allowing the force mediated, reversible unfolding of these domains, permitting extension and retraction of the external portions of these enormous proteins as they link to the surfaces of other cells [35]. This research used single molecule atomic force spectroscopy to show that application of a stretching force to one hPKD1 molecule resulted in the unfolding, one at a time, of the PKD repeats. Releasing the stretching force allowed the repeats to refold. The same phenomenon was found for the REJ domain of hPKD1, the data indicating that the unfolding of the REJ domain occurred in approximately 10 steps. Homology modeling suggests that there are 10 folded fibronectin-III domains within the REJ domain of hPKD1 [35]. In addition to a putative function in making SpREJ and PKD1 proteins capable of great elasticity, the REJ domain is also known to be required for cleavage of hPKD1 in the GPS domain, a process necessary to make a functional hPKD1 [28].

This study is the first to show the developmental expression of SpREJ in sea urchin embryos. SpREJ1 and 6 transcripts could not be found in embryos at our level of detection, indicating that these two proteins are probably not important for early development. SpREJ2 may be important during oogenesis, but remains at lower than unfertilized egg levels during development (Figure 2). SpREJ3 and 4 transcripts are also higher in eggs than in embryos. SpREJ5, 7, 9 and 10 transcripts show dramatic increases after 22 hours of development, suggesting that they are important for development of the gastrula, prism and pluteus stages. SpREJ8 transcripts are low in unfertilized eggs, increase to 22 hours and then decrease thereafter, suggesting that this protein is important for blastula formation. Future work on SpREJ proteins will involve *in situ *hybridization and antibodies to localize these transcripts and their proteins at different developmental stages.

RT-Q-PCR analysis of adult tissue expression of SpREJ transcripts indicates that, relative to the other SpREJs, SpREJ1 and 6 have dramatically high tissue-specific expression patterns (Figure 3). SpREJ6 is the only SpREJ with relatively low expression levels in testis. It was previously shown that SpREJ1 is localized in the plasma membrane covering the acrosome vesicle and flagella of sea urchin spermatozoa, but its expression in nongametic cells of the testis has not been studied. To generalize, except for SpREJ1 in testis, and SpREJ6 in muscle/test, most adult tissues contain easily detectable levels of SpREJ transcripts. Study of muscle/test preparations may reveal important functions of SpREJ proteins.

The sea urchin genome is 814 megabases (1), whereas the human is 3200 megabases. That the sea urchin has 10 *SpREJ *genes, while the human has five, shows either that humans lost ancestral PKD1 orthologs, or conversely, sea urchins gained new SpREJ genes. All five hPKD1 proteins have 11 TM, whereas only four of the SpREJ proteins have 11 TM. The unusual secondary structure predictions of SpREJ7-10 make them interesting candidate proteins for further study. SpREJ7 is odd in having six putative TM and a split REJ domain. SpREJ8 and 9 with only one putative TM, have no homology to any known domain upstream from their partial REJ domains. SpREJ9 also has the extremely Ser rich region close to the N-terminus. SpREJ10 is the oddest one in having a REJ domain, but no TM segments. Interestingly, all the genes encoding these proteins are transcribed in embryos or adult tissues. Continued studies of these novel SpREJ genes and their proteins may aid in discovering generalities about their functions.

## Conclusion

The sea urchin contains 10 SpREJ genes that are within the *PKD1 *gene family. All 10 are transcribed in either embryos or adult tissues. From amino- to carboxyl-termini, these SpREJ proteins usually contain: PKD repeats, a REJ domain, a GPS domain, a PLAT/LH2 domain and from 1–11 transmembrane segments. The predicted secondary structures of SpREJ7-10 are very different from other SpREJ/PKD1 proteins. Their future study may help define the multiply functions of the PKD1 family proteins.

## Methods

### Cloning and sequence analysis

The four known SpREJ cDNAs were used to search the sea urchin genome to find the six novel SpREJ gene models with REJ domains. Primers specific for each SpREJ were made using the six gene models and PCR reactions performed using *Strongylocentrotus purpuratus *testis, egg and embryo cDNAs as templates. The cDNA sequences were extended by 5' and 3' RACE (First Choice RLM kit, Ambion) and by gene walking with specific primers. PCR products were cloned into pCR 4-TOPO (Invitrogen, Carlsbad, California) and sequenced with gene specific primers. All cDNAs were prepared from total RNA by standard methods. The full-length sequences and start and stop codons of all 10 SpREJ cDNAs were experimentally determined. The copy number, length and number of exons/introns of each gene were determined using the *S. purpuratus *BAC plus WGS assembly version 2.0, of June 15, 2006 [36]. CLUSTALW (MacVector) was used to make nucleotide alignments. Domains were predicted from protein BLAST searches using the combination of NCBI [37] and EMBL-EBI [38] websites.

All sequences described in this paper have the following GenBank accession numbers: SpREJ1, NM214608; SpREJ2, NM214637; SpREJ3, NM214636; SpREJ4, AY620398; SpREJ5, DQ988048; SpREJ6, DQ988049; SpREJ7, DQ988050; SpREJ8, EF216328 ; SpREJ9, EF203419; and SpREJ10; EF203420. Human hPKD1 family GenBank accession numbers are: hPKD1, U24497; hPKDREJ, AF116458; hPKD1L1, NM138295; hPKD1L2, NM052892; and hPKD1L3, AY164485. Detailed annotation of *SpREJ *genes can be obtained from the sea urchin genome project website [39] (to access the *SpREJ *annotation page, select "common gene name", type "REJ" in the box and enter).

### Preparation of cDNAs from embryos and adult tissues

Adult tissues were separated by dissecting sea urchins and samples were dissolved in RLT buffer (RNAeasy Kit, Qiagen, Valencia, California). Gametes were spawned by injecting adults with 0.5 M KCl. Embryos were cultured at 0.25% v/v suspension, with constant 60 rpm stirring at 16°C in Millipore filtered seawater. Embryos were collected by hand centrifugation and 100 μl of packed embryo pellet dissolved in RLT buffer and total RNA extracted. RNA was quantified by absorption at 260 nm. Synthesis of cDNA was performed with 2 μg of total RNA using the SuperScript II polymerase and oligo (dT)12 -18 primers by standard procedures (Invitrogen) in a final volume of 20 μl.

### Real-time quantitative PCR

Forward (f) and reverse (r) primers were designed to amplify all 10 SpREJs cDNAs as follows (5' to 3'): SpREJ1f, CGATCTGGAAACAGAAGGAG and SpREJ1r, GATGGTTTGTGATGGACCAC (463 bp product); SpREJ2f, GGTCCATCACAAACCATCTATCAGG and SpREJ2r, GATTGGCTATGAAGCAGGAGTCC (306 bp product); SpREJ3f, TGGAGTTTGCTGCTGCTATGC and SpREJ3r, CGAACACTGTGCTTGACCATTG (272 bp product); SpREJ4f, TGACCGTCCTGGAGAGCATTAC and SpREJ4r, CCTGGGATTGAAAGGCAAGTC (224 bp product); SpREJ5f, TGTTCTACAGCAAGACCCGTCG and SpREJ5r, CCACGATGAGATTAGCAGGGAAC (279 bp product); SpREJ6f, ACCACTCATTGTCGTCATTAGCG and SpREJ6r, TGGCATCAAGTCTTAGGGCTCC (290 bp product); SpREJ7f, CAATCACACCTTTACGGAAGTTGG and SpREJ7r, GCTGATGGGCTCCTGCACTAACAG (382 bp product), SpREJ8f, CCCGACAGTGAAAATGAAGAGATG and SpREJ8r, CCAGACTTGGAAAGGATGGTGAC (251 bp product); SpREJ9f, TGCGAACAGACGGATGACAAC and SpREJ9r, CCTGAAGTGGTATCAACAGTGGC (350 bp product); SpREJ10f, TGAAGCAGTTGTTGTCTCCAGATG and SpREJ10r, CAGCATAGAGCAGAGGTAAGCAGTC (324 bp product); Ubi-f, CGAGTATTTGCCAGATGTGAACCC and Ubi-r, ATTGGATTTTTTGCCCCTGC (233 bp product) for Sp-ubiquitin (GenBank accession number M61772). These primer pairs were used to PCR amplify cDNA prepared from adult tissues and embryos. To construct standard curves, gel purified (Qiagen) template cDNA quantities were prepared by serial dilution. RT-Q-PCR was performed on a LightCycler II (Roche) instrument with LightCycler FastStart DNA Master^PLUS ^SYBR Green I reagents (Roche). Triplicate dilutions (5, 25 and 50-fold) (for embryonic expression experiments) and duplicate dilutions (1 and 10-fold) (tissue expression experiment) of template cDNA from each unknown were added at 5 μl/reaction (total volume 20 μl). The quantities of the unknowns, obtained in either triplicate or duplicate, were analyzed individually and then determined from the standard curve. Variations among developmental stages were corrected by normalization to ubiquitin. Adult tissue data were also normalized to ubiquitin with the caveat that the constancy of ubiquitin transcripts in adult tissues is unknown. The specificity of each RTQ-PCR product was assayed by a melting curve analysis and agarose gel electrophoresis.

## List of abbreviations

ADPKD, autosomal-dominant polycystic kidney disease; GPS, G-protein coupled receptor cleavage site; PKD, polycystic kidney disease; REJ, Receptor for Egg Jelly; hPKD1L, human PKD1-like; TM, transmembrane segment; TRP, transient receptor potential cation channel; Ubi, ubiquitin; Sp, *Strongylocentrotus purpuratus; *PLAT/LH2, polycystin-1, lipoxygenase, alpha-toxin/lipoxygenase homology 2 domain;

## Authors' contributions

All authors gathered the experiment data presented. HJG and VDV wrote the paper. All authors read the paper.
